# *Schistosoma mansoni* Egg-Released IPSE/alpha-1 Dampens Inflammatory Cytokine Responses *via* Basophil Interleukin (IL)-4 and IL-13

**DOI:** 10.3389/fimmu.2018.02293

**Published:** 2018-10-10

**Authors:** Katrin Knuhr, Kristina Langhans, Sandra Nyenhuis, Kerstin Viertmann, Anna M. Overgaard Kildemoes, Michael J. Doenhoff, Helmut Haas, Gabriele Schramm

**Affiliations:** ^1^Experimental Pneumology, Research Center Borstel, Airway Research Center North, Member of the German Center for Lung Research (DZL), Borstel, Germany; ^2^Section for Parasitology and Aquatic Pathobiology, Faculty of Health and Medical Sciences, University of Copenhagen, Copenhagen, Denmark; ^3^School of Life Sciences, University of Nottingham, Nottingham, United Kingdom; ^4^helminGuard, Süelfeld, Germany

**Keywords:** *Schistosoma mansoni*, IPSE/alpha-1, inflammation, basophils, Interleukin-4, Interleukin-13, alternatively activated macrophages, Toll-like receptor

## Abstract

Schistosomes control inflammation in their hosts *via* highly effective mechanisms such as induction of Tregs, Bregs, and alternatively activated macrophages (AAMs). Notably, IPSE/alpha-1, the major secretory product from *Schistosoma mansoni* eggs, triggers basophils to release interleukin (IL)-4 and IL-13. Both cytokines are essential for AAM induction, suggesting an important role for IPSE/alpha-1 in inflammation control. Here, we show by *in vitro* co-culture experiments that IPSE/alpha-1-induced basophil IL-4/IL-13 inhibited pro-inflammatory cytokine release from human LPS-activated monocytes. This effect was cell/cell contact-independent but dependent on IL-4, since it was abrogated in the presence of anti-IL-4 antibodies. Importantly, the IPSE/alpha-1-induced IL-4/IL-13 release from basophils was amplified in the presence of LPS. Moreover, monocytes co-cultured in the presence of LPS with IPSE/alpha-1-stimulated basophils adopted an AAM-like phenotype as assessed by elevated expression of CD206 and CD209. The putative *in vivo* relevance of these findings was supported by immunohistological staining of *S. mansoni*-infected murine tissue revealing close physical contact between IPSE/alpha-1 and basophils in schistosome egg granulomas. Taken together, we found that IPSE/alpha-1 dampens inflammatory cytokine responses by triggering basophil IL-4/IL-13, in particular in the context of TLR activation, thereby turning inflammatory monocytes into anti-inflammatory AAMs. This might represent a mechanism used by schistosomes to control inflammation in the host.

## Introduction

Helminths have strong modulatory effects on the immune system of their hosts (1). For example, epidemiological studies and animal experiments have shown that helminth infections can protect against asthma, allergies and autoimmune diseases (2). In particular, the trematode *Schistosoma mansoni* is a potent immunoregulator. Each schistosome couple produces approximately 300 eggs per day, which are laid in the mesenteric veins. About 50% of the eggs are carried away with the blood stream and embolize in the liver, where they induce granulomatous inflammation. The other 50% migrate from the blood vessels through the intestinal tissue toward the gut lumen. In the process they induce mucosal granulomatous inflammation, leaving behind phlegmonous migration channels (3, 4). The inflammation permits egg egress, maintaining the life cycle of the parasite (5, 6). However, the perforation channels enable bacteria to enter the gut wall. Given the high number of eggs (and consequent perforation channels), one would expect extensive intestinal inflammation (and subsequent symptoms) in infected individuals. However, most infected individuals develop only moderate intestinal symptoms, such as intermittent abdominal pain and diarrhea (3), suggesting that inflammation is kept tightly under control. Experiments with *S. mansoni*-infected mice revealed that IL-4 and intact IL-4 receptor signaling is essential for the protection of mice from intestinal inflammation, presumably *via* induction of alternatively activated macrophages (AAMs) (7–9). As a major source of IL-4 in helminth infections, including schistosomiasis, basophils have been identified (10–12).

Schistosome eggs have been shown to secrete proteins with putative immunomodulatory functions (13). The major secreted product is the glycoprotein IPSE/alpha-1, representing 80% of the egg secretions (14). Previously, we found that IPSE/alpha-1 triggers the release of IL-4 and IL-13 from human and murine basophils (15, 16), suggesting that this molecule is involved in inflammation control in schistosomiasis. IPSE/alpha-1 is a general immunoglobulin-binding factor that binds with highest affinity to the IgE isotype (17). Upon interaction with IgE bound to the FcεRI receptor on the surface of basophils, IPSE/alpha-1 activates basophils to release IL-4 and IL-13. Removal of IgE from the receptor (IgE stripping) abrogates the activation, while resensitization with IgE restores the cytokine releasing activity of IPSE/alpha-1 on basophils (16, 18). Noteworthy binding of IPSE/alpha-1 to IgE is independent of the IgE specificity (17). Structure analysis revealed that IPSE/alpha-1 belongs to the beta-gamma crystallin family (17). Moreover, IPSE/alpha-1 has a C-terminal functional nuclear localization sequence (NLS) for translocation into the nucleus (19). Natural IPSE/alpha-1 is N-glycosylated and contains Lewis X-motifs (20). Glycosylation is essential for binding to and uptake from monocyte-derived dendritic cells, presumably *via* lectin receptors (19). However, induction of IL-4 and IL-13 from basophils does not require uptake of IPSE/alpha-1 into the basophils and is independent of NLS and glycosylation (16, 19).

Recently, further immunomodulatory functions of IPSE/alpha-1 have been described: IPSE/alpha-1 was shown to be able to induce development of regulatory B cells (21). A mutant of IPSE/alpha-1, called SmCKBP (*S. mansoni* chemokine binding protein) was found to bind to chemokines (IL-8), thus neutralizing pro-inflammatory cytokines (22). Noteworthy, SmCKBP is not able to activate basophils. This is due to one of two amino acid exchanges (T92Y and R127L). T92 was mapped by NMR titrations in the putative IgE binding site of IPSE/alpha-1, which is important for interaction and subsequent activation of basophils (17).

Finally, an IPSE/alpha-1 homolog (named H-IPSE) was identified in *S. haematobium* (23). Also H-IPSE is able to translocate into the nucleus of cells *via* a functional nuclear localization sequence and to induce IL-4 release from murine basophils (24).

Given the importance of IL-4 and IL-4 signaling for host protection, and knowing that basophils are a source of IL-4, we asked in this study whether IPSE/alpha-1, as a potent inducer of basophil IL-4/IL-13, might be involved in the control of inflammation in schistosomiasis.

## Materials and methods

### Recombinant IPSE/alpha-1

IPSE/alpha-1 was expressed in *E. coli* and refolded as described in detail by Schramm et al. (16) The LPS content of the recombinant *E. coli*-expressed IPSE/alpha-1 preparation was 98 ng/mg protein as determined by Limulus amebocyte lysate (LAL) assay (Haemochrom). *E. coli*-expressed IPSE/alpha-1 is not glycosylated in contrast to the natural protein. However, the glycosylation is not involved in the basophil IL-4-inducing function. Natural and *E. coli*-expressed unglycosylated IPSE/alpha-1 induced comparable amounts of cytokines from human basophils (16).

### Isolation of human peripheral blood cells

Co-cultures were performed with monocytes and basophils obtained from the same donor (autologous system). Monocytes and basophils were isolated from 250 ml of peripheral blood from healthy donors. Ethylene diamine tetra-acetic acid (EDTA) blood was divided into two parts and separated *via* density gradient centrifugation, one half for isolation of monocytes *via* Ficoll (density 1.077 g/l), and the other half for isolation of basophils *via* Ficoll / Percoll (100/6, density 1.080 g/l). At a density of 1.080 g/l the interphase contains both mononuclear cells and basophilic granulocytes. The respective cell populations were then enriched *via* counter flow elutriation. Basophils were further purified by magnetic cell sorting (MACS) using the basophil isolation kit II for negative selection of basophils (Miltenyi-Biotech). The whole three-step protocol for basophil isolation was described by Haisch et al. (21). Across the samples, ≥98% purity was achieved. Purity was determined by microscopic assessment of May-Gruenwald-stained cytospins.

### Ethics statement

Peripheral blood was taken from adult healthy donors. All donors provided written informed consent (Ethics approval by the Ethics Committee of the University of Luebeck; AZ-12-202A). Experiments that used mice for obtaining mouse tissue for immunofluorescence stainings were approved by the Ethical Review Committee of the University of Nottingham in which these materials were produced and the work was carried out in strict accordance with UK government regulations for animal welfare and amelioration of suffering in force at the time. The work was licensed under legislation specified by the UK Animals (Scientific Procedures) Act 1986, (project license numbers PPL 40/3024 and 40/3595). Animals were killed by administration of a lethal dose of pentobarbitone anesthetic.

Experiments that used mice for obtaining mouse tissue for immunohistochemical stainings were performed according to the Danish Act on Animal Experimentation (LBK no. 474 of 15/05/2014). The study was approved by the Animal Experimentation Inspectorate, Ministry of Environment and Food, Denmark (license no. 2015-15-0201-00694).

### Cell culture and stimulation

Cell culture was performed in Iscove's Modified Dulbecco's Media (IMDM; PAA) containing 2 mM glutamine (PAA), 5 μg/ml insulin (Gibco), 50 μg/ml apo-transferrin (Sigma-Aldrich), 100 μg/ml Pen/Strep (PAA), 10% heat-inactivated Fetal Calf Serum (FCS-Gold; PAA) and 2.5 ng/ml IL-3 (kind gift of Kirin Brewery, Japan). IL-3 was added to all cultures containing basophils as a survival factor for basophils (25). For co-culture, autologous monocytes and basophils were incubated in a 1:1 ratio, i.e., 250,000 monocytes and 250,000 basophils in 1 ml culture medium per well in 24-well flat-bottom culture plates. Following purification, basophils were pre-incubated for regeneration for 30 min at 37°C, 6% CO_2_ before they were added to the monocytes. Stimuli were added to a final concentration as follows: 10 ng/ml LPS (*Salmonella friedenau*, kind gift of Prof. H. Brade, RCB), 120 pg/ml recombinant human IL-4 (rhIL-4) (Immunotools), 100 ng/ml recombinant IPSE/alpha-1. Cells were co-cultured for 24 h, unless specified otherwise, and were harvested and analyzed at the flow cytometer LSR II (BD Biosciences). Stimulation of basophils was additionally performed with 50 ng/ml goat anti-human IgE (Dianova) as positive control for IgE receptor-mediated stimulation, and with 2 μM ionomycin for induction of maximal cytokine release. All stimulations of basophils, except with ionomycin, were performed in the presence of IL-3 at a concentration of 2.5 ng/ml for induction of IL-4 release and at a concentration of 0.1 ng/ml for induction of IL-13 release, since higher concentrations of IL-3 *per se* induce IL-13 release from basophils. Supernatants were kept at −80°C until analyzed for cytokine production by the respective cytokine-specific ELISAs: IL-1β and IL-6 from monocytes (BD Biosciences); IL-4 and IL-13 from basophils (Diaclone). Measurement was performed using the ELISA-Reader SunRise (Tecan).

### Analysis of expression of surface markers on monocytes and TLR on basophils by flow cytometry (LSRII)

Expression of the surface markers CD206 and CD209 on monocytes and TLR1, 2, 4, and 6 on basophils was analyzed by flow cytometry following staining with specific fluorescent-labeled antibodies. Monocytes were stained with FITC-anti-human CD206 and PE-anti-human CD209, obtained from BD Biosciences. Basophils were stained with biotin-labeled-anti-TLR1 followed by PerCP-streptavidin, AF647-labeled anti-TLR2, BV421-labeled anti-TLR4, and PE-labeled-anti-TLR6, respectively, obtained from Biolegend. Staining was performed in 96-well V-bottom plates with 50,000 monocytes or 125,000 basophils per well in 20 μl of the respective antibodies diluted 1:20 in FACS staining buffer (10% FCS, 1% HS, 0.5% BSA in PBS). For control, cells were left unstained, and single stained (CD206, CD209). For compensation controls, we used compensation beads (BD CompBead Plus, BD Biosciences). Measurement was performed on the BD LSRII cytometer and data were analyzed with DIVA 8.0 software (BD Biosciences).

### Immunohistology

Sections of *S. mansoni*-infected or uninfected paraffin-embedded mouse tissues [liver and intestine/ileum from random-bred CD1 mice (Charles River, UK)] were stained for basophils with the basophil-specific rat anti-mMCP-8 antibody (clone TUG8, Biolegend), either by immunofluorescence or immunohistochemically. For immunofluorescence, anti-mMCP-8 was diluted 1:500. Binding was detected by AF-633-labeled secondary rabbit anti-rat IgG (Invitrogen), dilution 1:1000. Nuclei were counterstained by DAPI (Invitrogen; dilution 1:1,000), and pictures were taken with an Olympus IX-81 inverse fluorescence microscope (Olympus). Sections of *S. mansoni*-infected paraffin-embedded mouse liver tissue from female C57BL/6-NTAC mice (Taconic, Denmark) were immunohistochemically stained the anti-mMCP-8 and the secondary biotinylated rabbit anti-rat antibody were diluted 1:100, and detection was performed using the Vectastain Kit (Vector Laboratories). For detection of IPSE/alpha-1 the monoclonal antibody anti-IPSE/alpha-1 (clone 74 1G2) (16), was biotinylated according to standard protocols and diluted 1:100. To prevent non-specific binding to the mouse tissue, the M.O.M. Peroxidase kit (Vector laboratories) was applied. Mayer‘s hematoxylin (Merck) was used for counter staining. Pictures were taken at the Olympus BX51 microscope.

### Isolation of RNA from basophils and analysis of TLR expression by RT-PCR with TLR-specific primers

RNA was isolated from basophils with a purity ≥99% using the “Direct-zol RNA MiniPrep Kit” from Zymo Research and reversely transcribed into cDNA using the “RevertAid H Minus First Strand cDNA synthesis Kit” (Thermo Scientific). A minimum of 5 × 10^6^ basophils was required to obtain sufficient amount of RNA. The RNA was either stored at −80°C or subjected immediately to the reverse transcription reaction. DNA concentration and quality was controlled with the Nanodrop photometer (Peqlab). Specific TLR cDNA was amplified by the following PCR protocol: pre-denaturation step at 95°C for 15 min followed by 40 cycles of denaturation at 94°C for 30 s, annealing at 55°C for 30 s, extension at 72°C for 60 s, followed by a final extension step at 72°C for 10 min before cooling to 4°C. Amplification was performed using the Maxima Hot Start DNA Polymerase (Thermo Scientific). Table 1 shows the sequences of the applied TLR-specific primers. A negative control reaction was performed without cDNA template and a positive control using specific primers for the housekeeping gene GAPDH. PCR products were analyzed on 1% agarose gels together with the “MassRuler DNA Ladder Mix” from Thermo Scientific (marker 1) and the “100 bp ladder” from Promega (marker 2).

**Table 1:** GAPDH and TLR-specific primer sequences.

	**Sequence forward**	**Sequence reverse**	**bp**
GAPD	5′-TGATGACATCAAGGTGGTGAAG-3′	5′-TCCTTGGAGGCCATGTGGGCCAT-3′	240
TLR1	5′-TATTGGGCACCCCTACAAAA-3′	5′-CCAATTCCTGGTTGAATTTGA-3′	375
TLR2	5′-GGGTCTTGGGGGTCATCATCA-3′	5′-CAAGACTGCCCAGGGAACAAAAAC-3′	282
TLR3	5′-AGGATTGGGTCTGGGAACAT-3′	5′-GCCAGTTCAAGATGCAGTGA-3′	339
TLR4	5′-AGAACTGCAGGTGCTGGATT-3′	5′-TCATAGGGTTCAGGGACAGG-3′	413
TLR5	5′-TTAAACAACCAGGGACCCTCT-3′	5′-TAAGGTTGGGCAGGTTTCTG-3′	517
TLR6	5′-TCACAATTCAGTTTCCACC-3′	5′-CACAGTCACAGCCAACACC-3′	296
TLR7	5′-TGCTCTGCTCTCTTCAACCA-3′	5′-TTTGACCCCAGTGGAATAGG-3′	377
TLR8	5′-TGCTGCAAGTTACGGAATGA-3′	5′-ATTTTGCAGCCCTTGAAATG-3′	371
TLR9	5′-GAAGGGACCTCGAGTGTGAA-3′	5′-AAGTGGGGCACAGACTTCAG-3′	255
TLR10	5′-GAGAAGCTGGCAACATGTCA-3′	5′-CTTTCTTCTGGCAGCTCTGG-3′	495

### Statistical analysis

Statistical analysis was performed using the Graph Pad Prism6 software (GraphPad Software, San Diego, CA). Since human primary immune cells have a highly variable individual reactivity, the co-culture data were normalized and analyzed using Wilcoxon matched-pairs signed rank test or paired *t*-test. To determine significance between three or more data sets, the two way ANOVA multiple comparison test was performed as indicated in the figure legends. A *p*-value smaller than 0.05 was considered statistically significant. Data are presented as ±SEM and sample sizes are given in the figure legends.

## Results

### IPSE/alpha-1-induced basophil IL-4 and IL-13 inhibited pro-inflammatory cytokine release from LPS-activated monocytes *in vitro*

**Figure 1:**
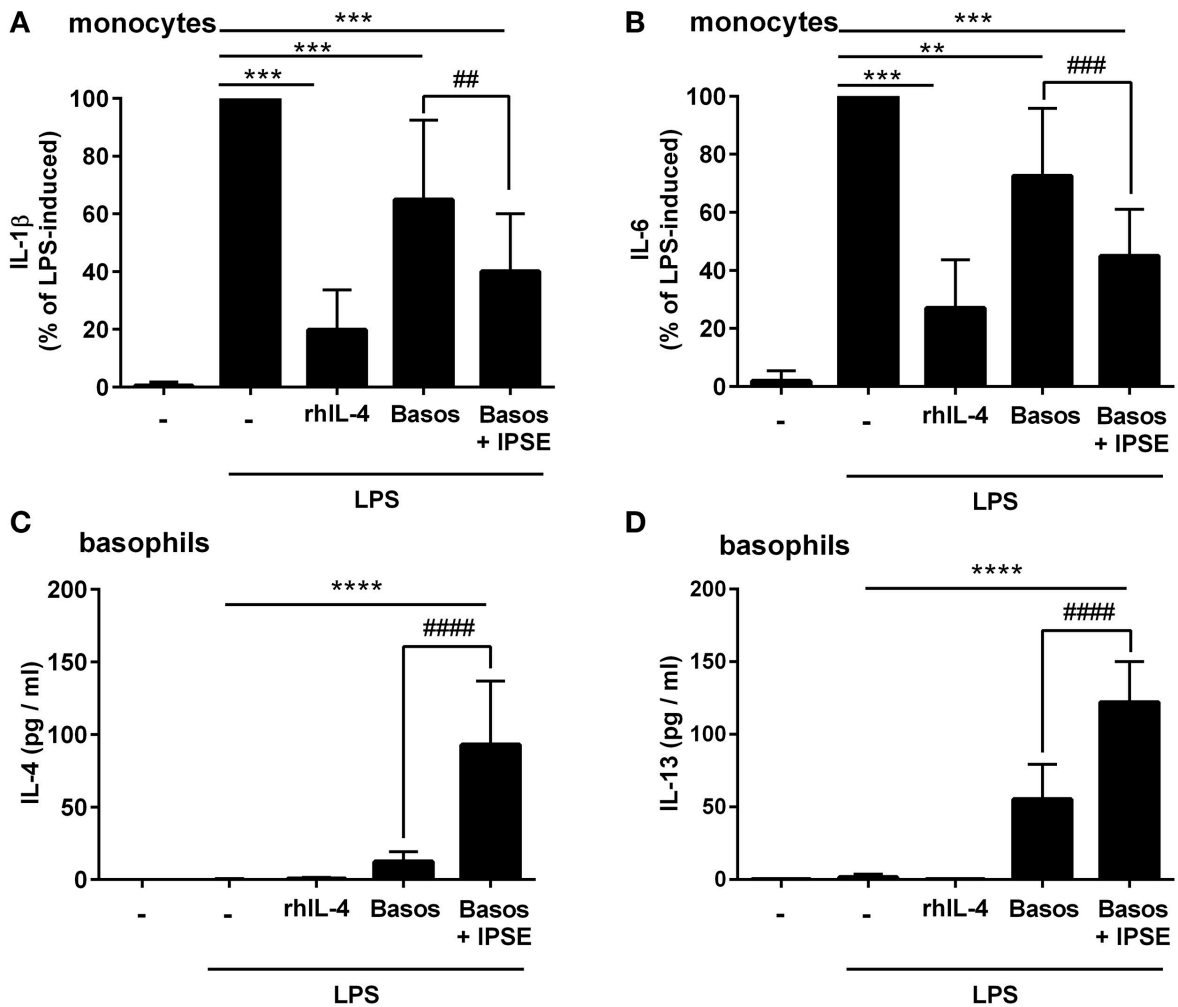
IPSE/alpha-1-triggered basophil IL-4 inhibited the release of IL-1β and IL-6 from human LPS-activated monocytes. Release of IL-1β **(A)** and IL-6 **(B)** from monocytes, and IL-4 **(C)** and IL-13 **(D)** from basophils. Monocyte-derived cytokine release **(A,B)** was normalized to the maximum release following LPS activation (100%). Note, rhIL-4 was only very poorly detected by the IL-4 ELISA (Diaclone), because the monoclonal antibodies in this ELISA are directed against natural IL-4 and do not bind properly to the recombinant IL-4. Data are presented as mean ± SEM, IL-1β and IL-6: *n* = 14; IL-4: *n* = 13; IL-13: *n* = 11. Statistics were performed with the Wilcoxon matched-pairs signed rank test **(A,B)** or paired *t*-test **(C,D)**; ^*^ = significance “LPS + additions (IL-4, basophils, basophils + IPSE)” vs. “LPS without additions”; ^**^p < 0.01, ^***^p < 0.001, ^****^p < 0.0001; ^#^ = significance “basophils with IPSE/alpha-1” vs. “without IPSE/alpha-1”; ^##^*p* < 0.01, ^###^*p* < 0.001, ^####^*p* < 0.0001.

**Figure 2:**
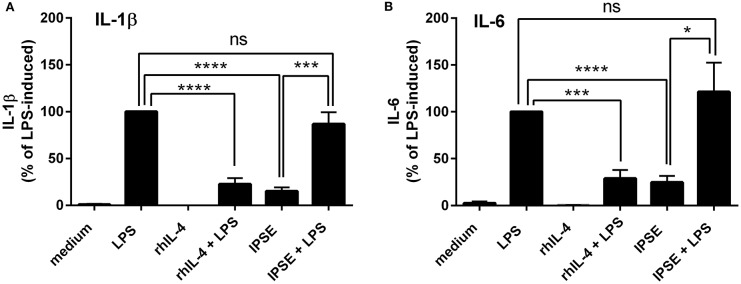
IPSE/alpha-1 had limited direct effect on the pro-inflammatory cytokine release of monocytes. IL-1β **(A)** and IL-6 **(B)** released from human monocytes stimulated with LPS, rhIL-4, IPSE/alpha-1 or combinations thereof. While rhuIL-4 had a significant inhibitory effect on the LPS-induced IL-1-β and IL-6 release from monocytes, IPSE/alpha-1 alone had only very little (IL-1β) or virtually no (IL-6) direct effect on LPS-activated monocytes with respect to inhibition of cytokine release. Pro-inflammatory cytokine release directly induced by IPSE/alpha-1 is the result of small amounts of LPS in *E. coli*-expressed IPSE/alpha-1. Data are presented as mean ± SEM, *n* = 7. Statistics were performed with the paired *t*-test; ns, not significant, ^*^p < 0.05, ^***^p < 0.001, ^****^p < 0.0001.

Human monocytes were isolated from peripheral blood of healthy donors, and were co-cultured with autologous basophils in a ratio of 1:1 in the presence of LPS, with or without IPSE/alpha-1. As control, monocytes were cultured without basophils in medium alone, and stimulated with LPS ± recombinant human IL-4 (rhIL-4). Figure 1 shows the results of 14 individual experiments. Monocytes released large amounts of the pro-inflammatory cytokines IL-1β and IL-6 upon activation with LPS. Since monocytes from individual human blood donors exhibited high variability in cytokine release, values were normalized by defining maximum LPS-induced cytokine release as 100%. The pro-inflammatory cytokine production of monocytes was significantly inhibited when exogenous recombinant human IL-4 was added. Co-culture with basophils alone without additional stimuli led to an inhibition in the production of IL-1β and IL-6. This inhibition was significantly enhanced when IPSE/alpha-1 was added to the co-culture (Figures 1A,B). Notably, the inhibitory effect significantly correlated with the amount of IL-4 and IL-13 released from the basophils (Wilcoxon matched-pairs signed rank test *p* < 0.001; Figures 1C,D). Since IPSE/alpha-1 is a general immunoglobulin-binding factor (17), with the potential to bind to receptors with immunoglobulin-like domains on the surface of cells and thus, could activate or inhibit their functions, we investigated the effect of IPSE/alpha-1 by itself (i.e., in the absence of basophils) on monocytes. We found that IPSE/alpha-1 did not inhibit, but—on the contrary—slightly induce pro-inflammatory cytokine release. This may be due to small amounts of contaminating LPS impurities in the recombinant IPSE/alpha-1 preparations from *E. coli* (Figure 2).

### Inhibition of pro-inflammatory cytokine release from LPS-activated monocytes by IPSE/alpha-1-stimulated basophils depended on soluble IL-4 and led to an “alternatively activated” phenotype of the monocytes

**Figure 3:**
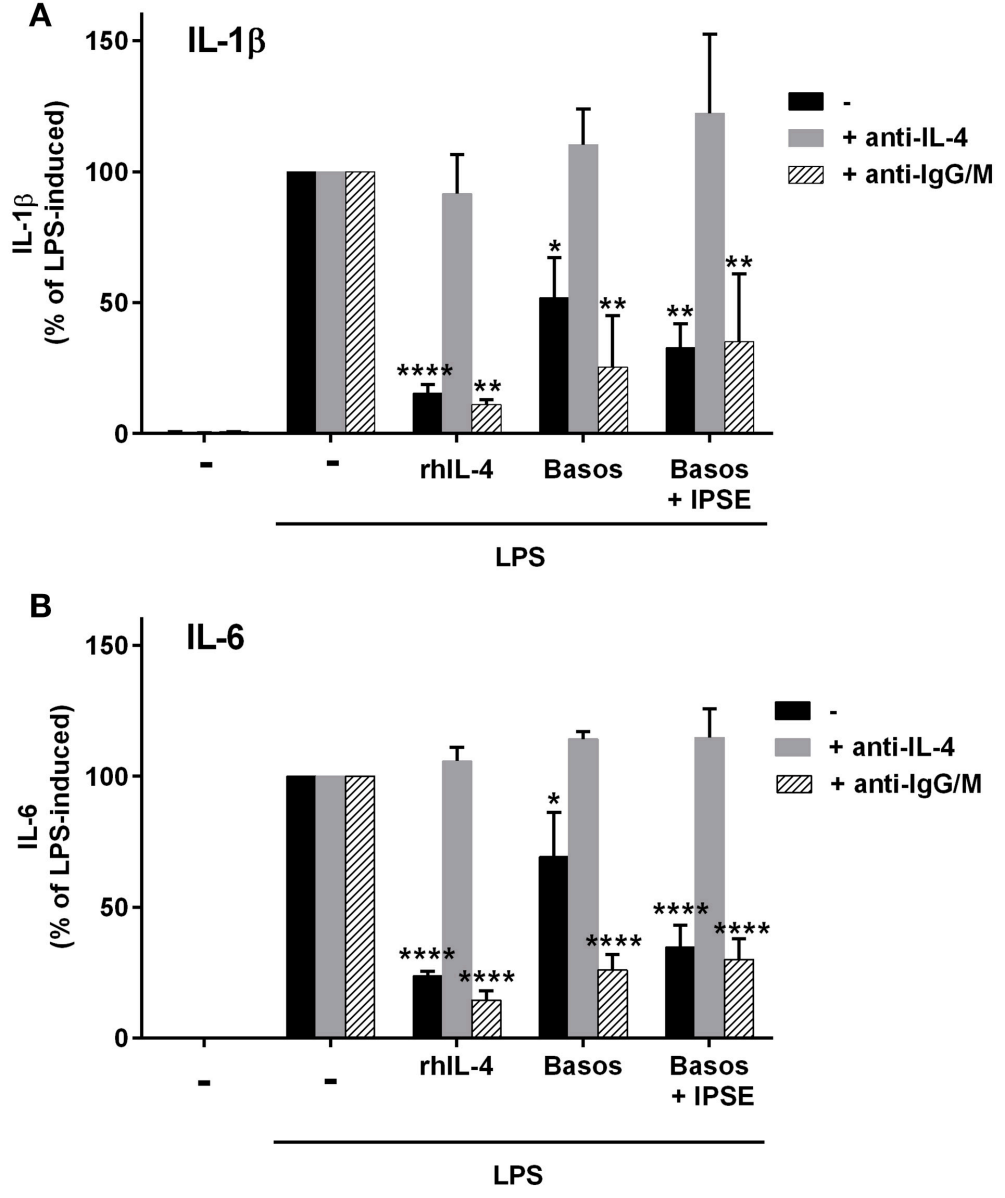
Anti-IL-4 abrogated the inhibitory effect of IPSE/alpha-1-triggered basophil-derived IL-4. Release of IL-1β **(A)** and IL-6 **(B)** from monocytes co-cultured with autologous basophils without (black bars) or with anti-IL-4 antibodies (gray bars) or with anti-IgG/M (bars with pattern) as control antibody. Inhibition of both IL-1β and IL-6 from LPS-activated monocytes was abrogated by anti-IL-4, while anti-IgG/M had no effect on the inhibition. Data were normalized to the maximum release following LPS activation (100%). Data are presented as mean ± SEM, *n* = 4 for anti-IL-4 and *n* = 2 for anti-IgG/M. Statistics were performed by two way ANOVA multiple comparison test; ^*^ = significance “LPS + additions (IL-4, basophils, basophils + IPSE)” vs. “LPS alone”; ^*^p < 0.05, ^**^p < 0.01, ^****^p < 0.0001.

**Figure 4:**
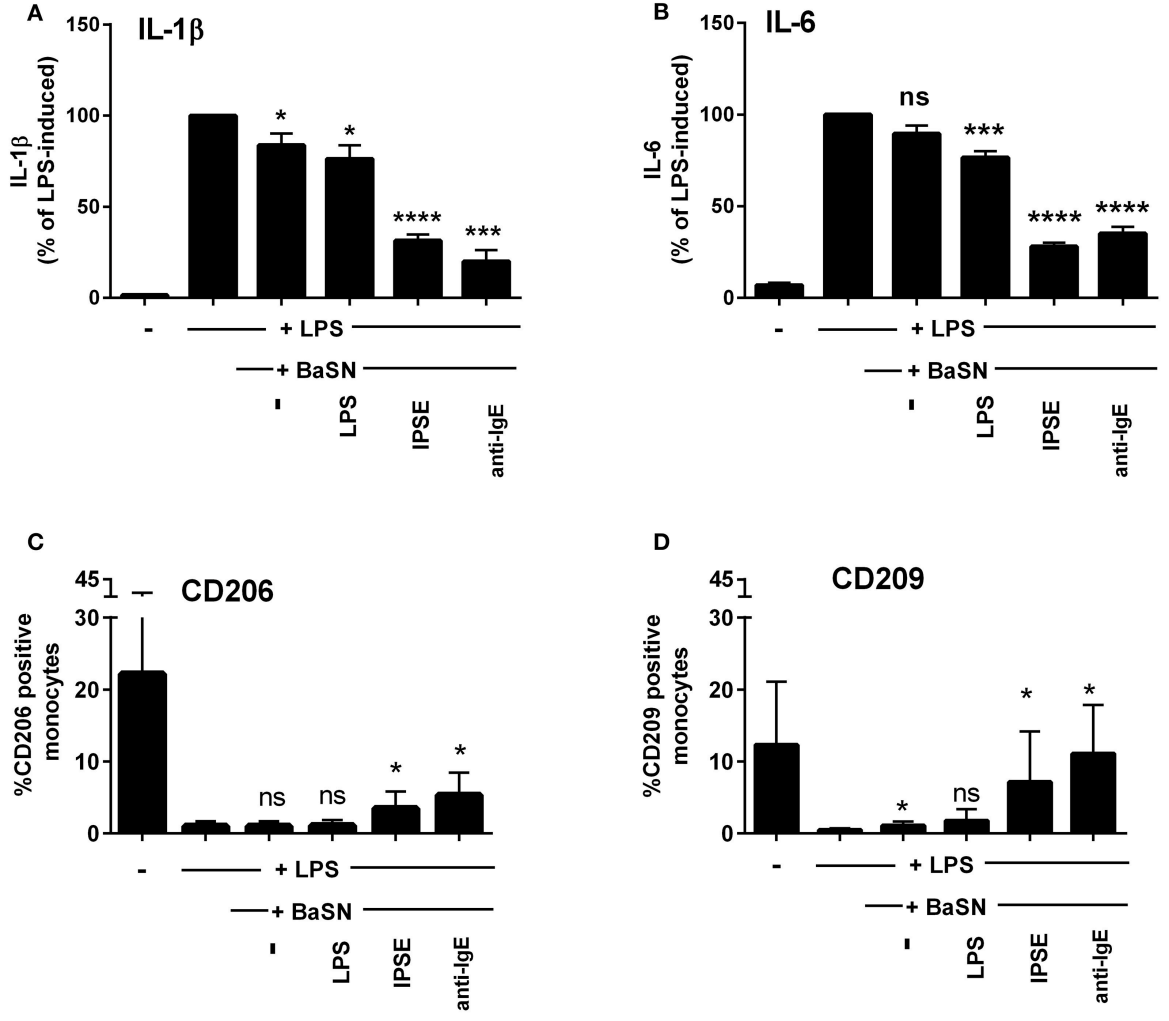
Culture of LPS-activated monocytes in supernatant from IPSE/alpha-1-stimulated basophils resulted in inhibition of pro-inflammatory cytokine release and increased numbers of CD206- and CD209-positive monocytes. IL-1β **(A)** and IL-6 **(B)** release from monocytes, and CD206 **(C)** and CD209 **(D)** expression on monocytes cultured with supernatant from basophils (BaSN). Monocyte-derived cytokine release was normalized to the maximum release following LPS activation (100%). Data are presented as mean ± SEM, *n* = 9 (anti-IgE: *n* = 5). Statistics were performed by paired *t*-test; significance was shown for “LPS with BaSN” vs. “LPS without BaSN” (black bar); ^*^p < 0.05, ^***^p < 0.001, ^****^p < 0.0001.

To demonstrate that basophil-derived IL-4 was responsible for the inhibition of pro-inflammatory cytokine release from LPS-activated monocytes, the co-culture described in the previous section was repeated in the presence of anti-IL-4 antibody and, as a negative control, an anti-IgG/M antibody with irrelevant specificity. Inhibition was completely abrogated in the presence of anti-IL-4, whereas addition of anti-IgG/M had no effect (Figure 3). Moreover, pro-inflammatory cytokine release was inhibited by culturing LPS-activated monocytes in basophil supernatant (BaSN), with the basophils stimulated before with IPSE/alpha-1 or anti-IgE (Figures 4A,B). Likewise, addition of anti-IL-4 antibodies abrogated also the inhibitory effect of supernatant of IPSE/alpha-1-stimulated basophils (data not shown). Taken together, these experiments confirm that inhibition of pro-inflammatory cytokine release from LPS-activated monocytes was IL-4-dependent, but cell/cell contact-independent.

Since IL-4 and IL-13 induce AAMs, we investigated the monocytes cultured in basophil-conditioned medium for the expression of CD206 (mannose receptor) and CD209 (DC-SIGN). Human monocytes freshly isolated from peripheral blood did not express CD206 or CD209 (not shown). Figures 4C,D show, that following culture in medium for 24 h, the percentage of CD206- and CD209-expressing monocytes increased to 22 and 12%, respectively. In the presence of LPS, only slight increase in CD206 or CD209 expression was observed (up to 1.9 and 0.8%, respectively). However, culturing LPS-activated monocytes in the supernatant of IPSE/alpha-1- or anti-IgE-stimulated basophils, elevated the expression of CD206 and CD209 up to 5 and 11%, respectively (Figures 4C,D). These results indicate that IPSE/alpha-1-induced IL-4 from basophils induced an alternatively activated-like phenotype in human monocytes.

### Basophils and IPSE/alpha-1 were detected in *S. mansoni* egg granulomas

Schistosome eggs deposited in host tissue attract a variety of immune cells, resulting in the formation of a granuloma around the eggs (4). The granuloma provides a defined area for close contact and interaction between egg-secreted products and host immune cells. Typically, schistosome eggs induce a Th2-granuloma, containing Th2 cells, B cells, eosinophils and AAMs (4). The amounts of IL-4 needed to create such a Th2-polarized immune response and induce AAMs might be provided by stimulation of basophils by egg-secreted IPSE/alpha-1. Thus, we investigated whether basophils are present in the granuloma, and whether they are in close contact with IPSE/alpha-1.

**Figure 5:**
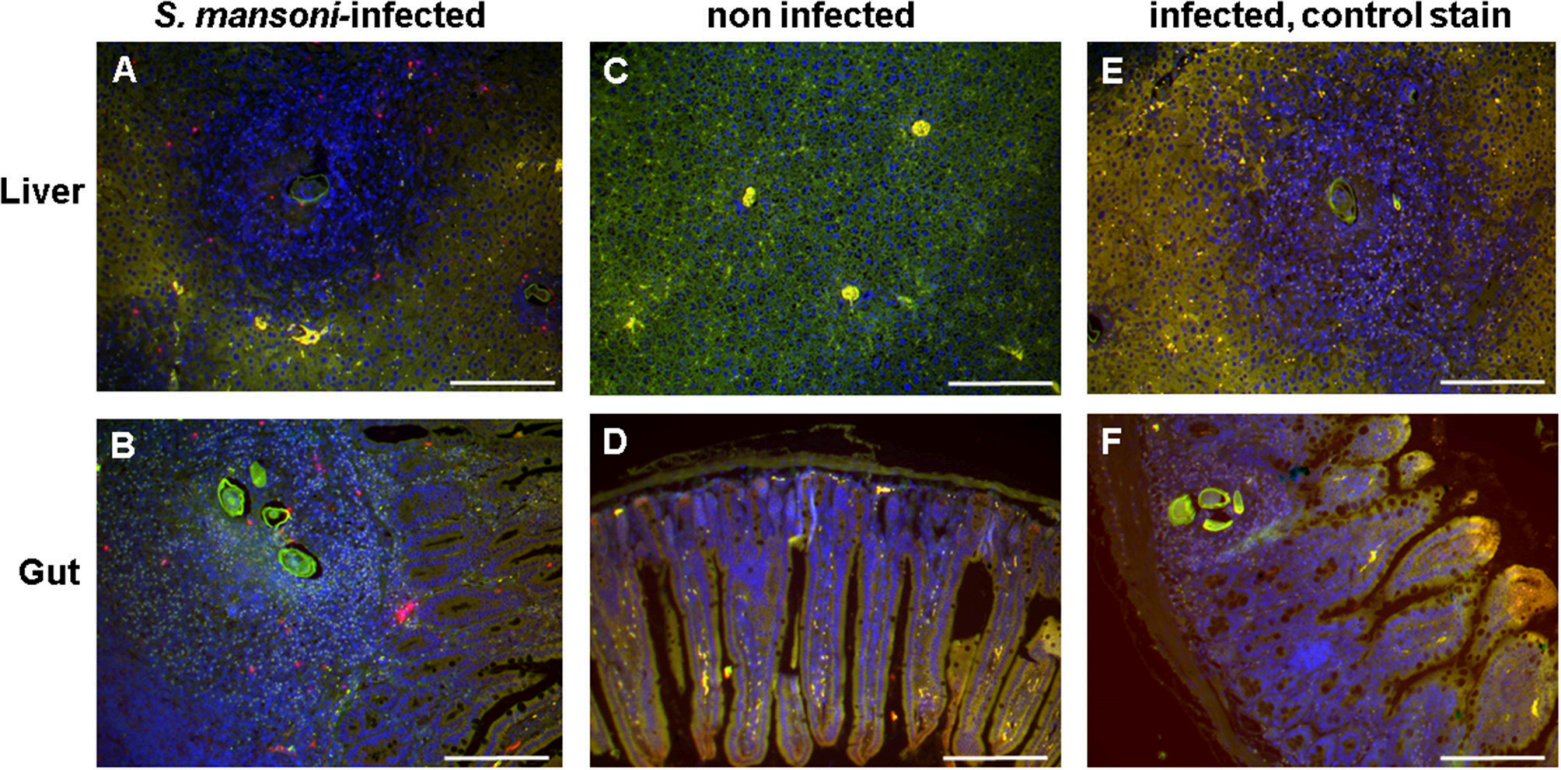
Basophils were detected in the egg granulomas in tissue of *S. mansoni*-infected mice. Liver **(A,C,E)** and gut (ileum) **(B,D,F)** of *S. mansoni*-infected mice **(A,B,E,F**) and uninfected control mice **(C,D)**; Nuclei are shown stained in blue; basophils (stained in magenta) were detected exclusively in *S. mansoni*-infected mouse tissue, i.e., in the granulomas. Note the autofluorescence of the schistosome egg shell (green). **(E,F)** are negative controls without the primary antibody anti-mMCP-8. Scale bars are 200 μm.

Sections of *S. mansoni*-infected mouse liver and gut (ileum) were stained with the basophil-specific antibody rat anti-mouse mast cell protease (mMCP)-8 and detected by fluorescence microscopy following incubation with the detection antibody AF633-labeled rabbit anti-rat IgG (Figure 5). Basophils (stained in magenta) could be clearly detected in the granulomas around the eggs, but not in the other areas of the infected tissues or in tissue of uninfected mice.

**Figure 6:**
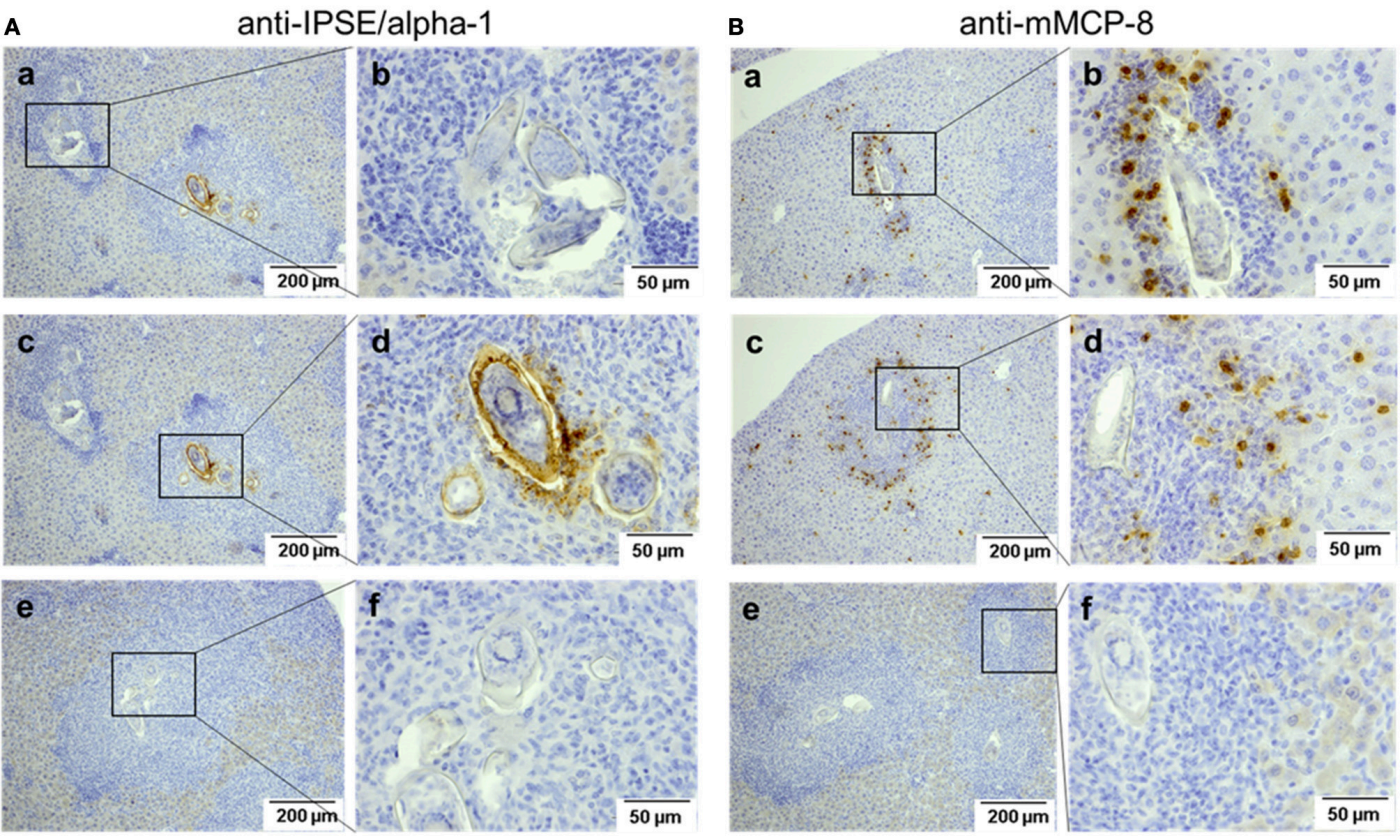
Detection of IPSE/alpha-1 and basophils in schistosome egg granulomas. Sections of *S. mansoni*-infected murine liver were stained for IPSE/alpha-1 (anti-IPSE/alpha-1; **A**) and basophils (anti-mMCP-8; **B**). Depicted are two magnification, each. Scale bars are 200 μm for 100x magnification and 50 μm for 400x magnification. (a,b) Show small granulomas with presumably immature eggs without IPSE/alpha-1 production **(A**a, left box, **A**b**)** and with basophils detected near the egg **(B**a,**B**b**)**. **(c,d)** Show advanced granulomas with strong IPSE/alpha-1 staining in the subshell area and outside the eggs **(A**c right box, **A**d**)** and with basophils detected mainly in the outer rim of the granuloma **(B**c,d**)**. (e,f) Show negative controls without the respective primary antibodies.

IPSE-alpha-1 is the major excretory/secretory product from mature eggs of *S. mansoni* (14). Thus, it would be expected to come into close contact with immune cells, i.e., with basophils in the granuloma surrounding the egg during its migration through the tissue. We stained sections of liver from *S. mansoni*-infected mice with the biotinylated anti-IPSE/alpha-1 monoclonal antibody 74-1G2 for IPSE/alpha-1 (Figure 6A) or with the anti-mMCP-8 antibody and a biotinylated rabbit anti-rat IgG for basophils (Figure 6B). Detection was performed immunohistochemically by streptavidin-labeled peroxidase. Both, IPSE/alpha-1 and basophils were detected in the granulomas. Eggs differed in their expression of IPSE/alpha-1, presumably representing different maturation stages (i.e., times after egg deposition). Figures 6Aa, left box and Ab show eggs, which do not express IPSE/alpha-1, presumably freshly deposited eggs in early granulomas, while Figures 6Ac, right box and Ad show advanced granulomas with pronounced IPSE/alpha-1 staining in the subshell area and outside the eggs.

Basophils were detected in small granulomas close to the eggs (Figures 6Ba,Bb), but in large granulomas mainly in the outer rim (Figures 6Bc,Bd).

### IgE-dependent cytokine release from basophils was boosted by simultaneous TLR ligation

Upon release of schistosome eggs into the gut lumen, granuloma cells come into contact with bacteria and bacterial products, and thus ligands for Toll-like receptors (TLRs). To assess the effect of LPS on basophils present in the granuloma, we investigated the expression of TLRs on basophils, and the release of IL-4 and IL-13 following simultaneous activation of basophils *via* the TLR and Fcε receptor (FcεRI).

To analyze the expression of TLR by basophils, we isolated mRNA from human basophils purified from peripheral blood of 12 individual healthy donors, and performed reverse transcription-polymerase chain reaction (RT-PCR) with TLR-specific primers.

**Figure 7:**
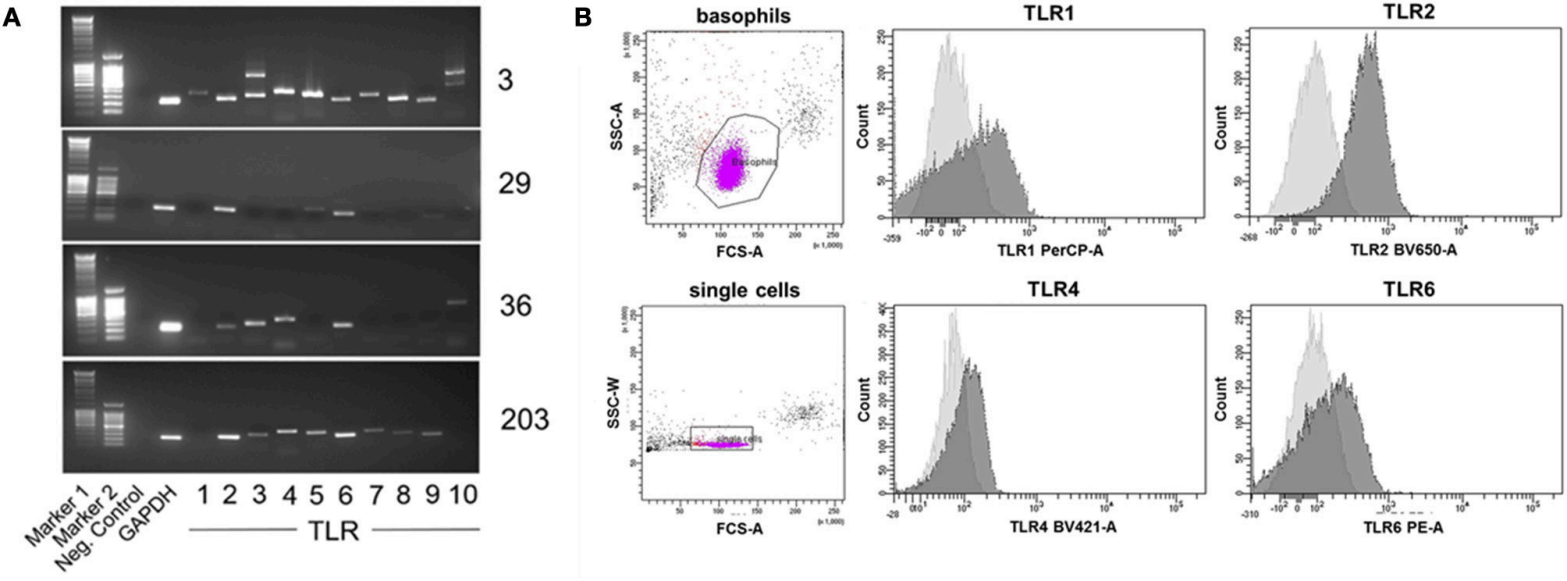
Individual TLR expression on human basophils as assessed by RT-PCR and surface staining followed by flow cytometry. **(A)** Expression of TLR1−10 cDNA as assessed by RT-PCR of four individual donors shown as an example for distinct expression patterns. The full set of donors are listed in Table 2. **(B)** Expression of the surface-exposed TLR1, 2, 4, and 6 on basophils of donor 29 measured by flow cytometry. Depicted are the histogram overlays of unstained (light gray) and with the respective fluorescently-labeled antibody stained (dark gray) basophils.

TLR expression differed in individual donors (see Figure 7A for expression of TLR1 to TLR10 mRNA in basophils of four out of 12 investigated donors as examples of distinct TLR expression patterns, and Table 2 for TLR expression of all investigated donors). We did not observe differences between allergic and non-allergic individuals or so-called “releasers” and “non-releasers” (donors whose basophils release or do not release histamine upon stimulation with anti-IgE).

**Table 2:** Individual TLR expression on human basophils as assessed by RT-PCR with TLR-specific primers.

**Donor**	**TLR1**	**TLR2**	**TLR3**	**TLR4**	**TLR5**	**TLR6**	**TLR7**	**TLR8**	**TLR9**	**TLR10**
2		+	+	+	+	+			+	
3	+	+	+	+	+	+	+	+	+	+
4	+	+	+	+	+	+		+	+	
8		+		+		+		+	+	+
13		+	+	+	+	+			+	+
26		+	+			+			+	+
29		+			+	+			+	
36		+	+	+		+				
184	+	+	+	+	+	+			+	+
200		+			+	+	+	+		
203		+	+	+	+	+	+	+	+	
224		+	+	+		+			+	

*TLR 2 and TLR 6 were detected on all investigated donors (dark gray shaded). Light gray-labeled donors are shown as examples of distinct TLR expression patterns in Figure 7A*.

To verify these results in terms of the effect on protein levels, we stained basophils of one example donor 29 with fluorescence-labeled antibodies against the surface-expressed TLR1, 2, 4, and 6, with analysis performed by flow cytometry (Figure 7B). Consistent with the RT-PCR results, basophils of this donor expressed TLR2 and TLR6 but only little TLR1 and TLR4.

**Figure 8:**
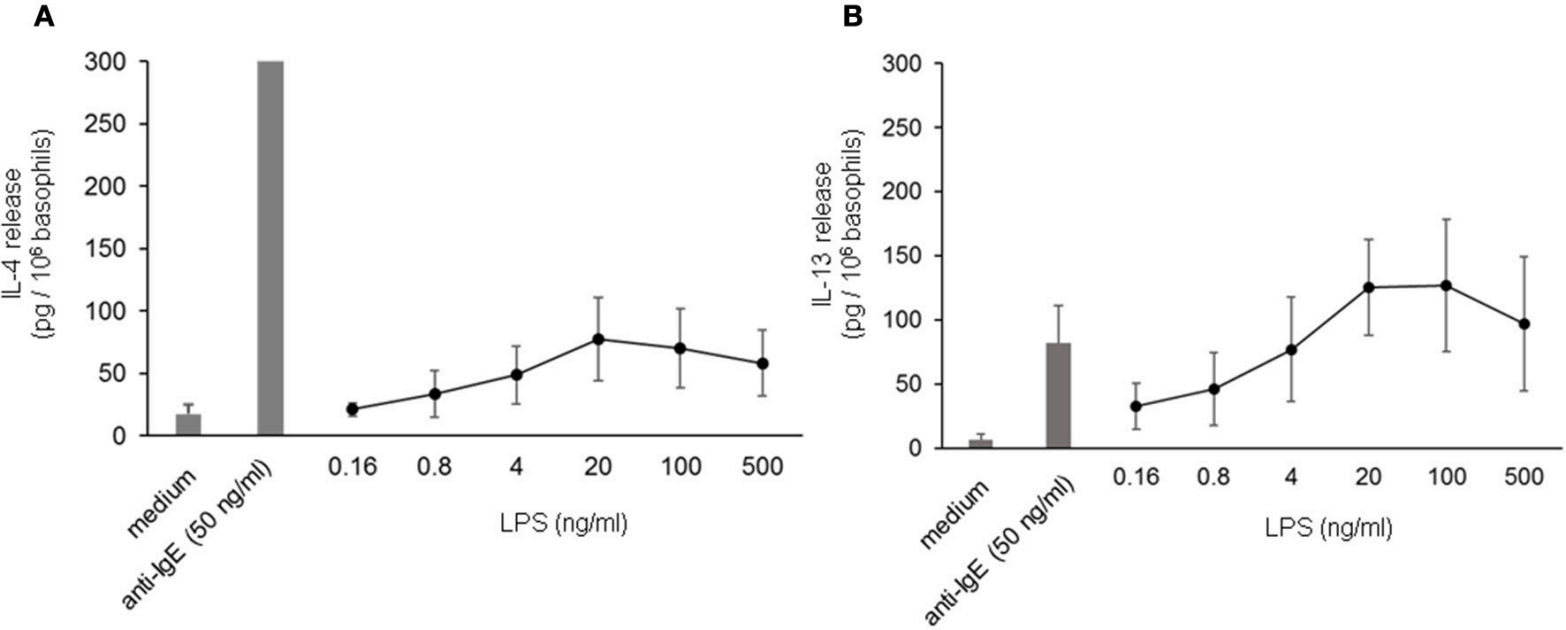
IL-4 and IL-13 release from basophils following stimulation with LPS. IL-4 **(A)** and IL-13 **(B**) release from basophils stimulated with different concentrations of LPS in comparison to anti-IgE. All stimulations were performed in the presence of IL-3. Data are presented as mean ± SEM; **(A)** *n* = 7, **(B)** *n* = 5.

**Figure 9:**
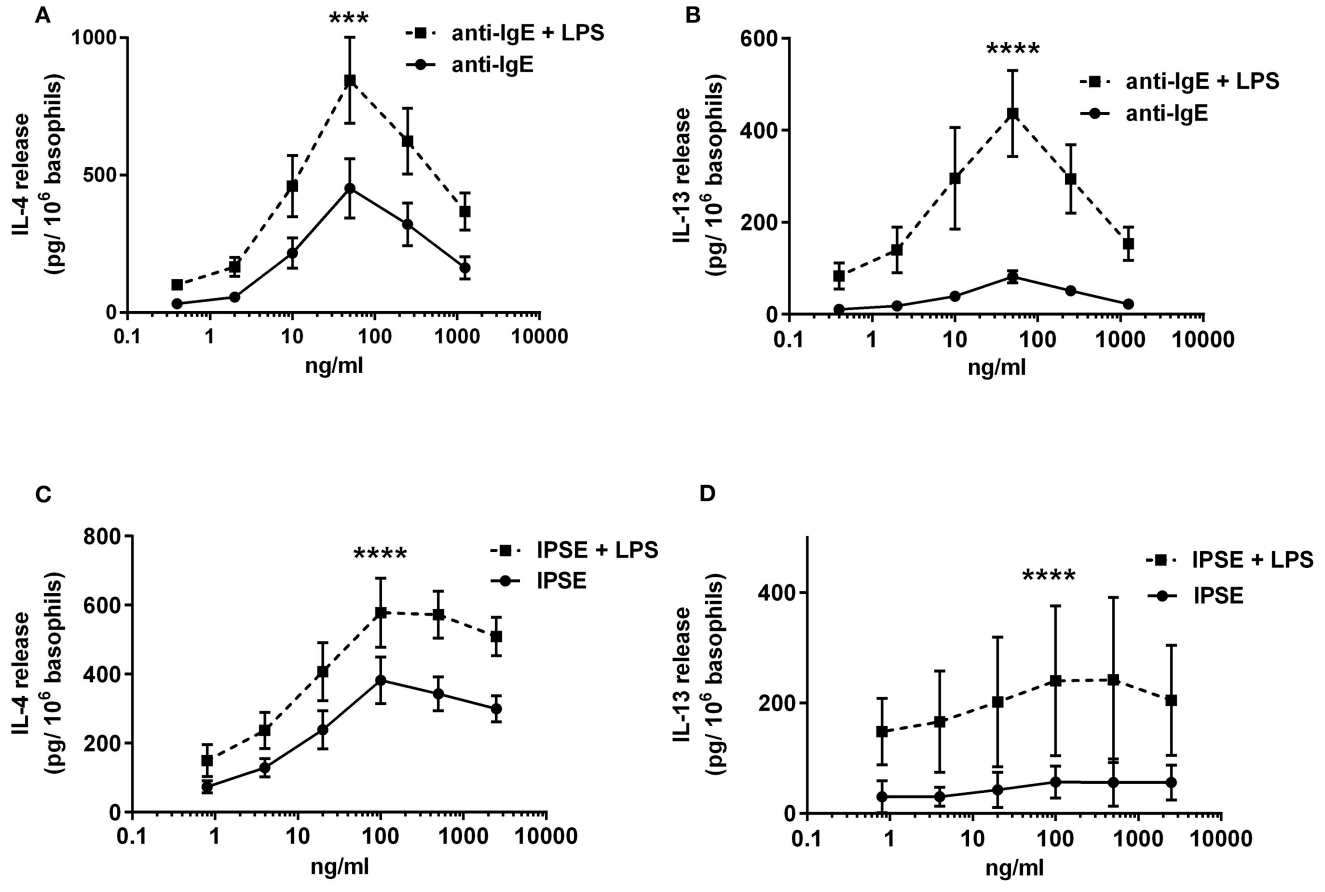
Enhanced FcεRI-dependent IL-4 and IL-13 release from basophils by simultaneous stimulation with LPS (*via* TLR4). IL-4 **(A,C)** and IL-13 **(B,D)** release from basophils stimulated anti-IgE ± LPS **(A,B)**, or IPSE/alpha-1 ± LPS **(C,D)**. Data are presented as mean ± SEM; **(A)** *n* = 7; **(B)** *n* = 5; **(C)** *n* = 9; **(D)** *n* = 4. Statistics data were performed by ratio paired *t*-test; significance is shown as with vs. without LPS; ^***^p < 0.001, ^****^p < 0.0001.

To investigate the influence of LPS on IgE-receptor-mediated cytokine release from basophils, purified basophils were stimulated with LPS, anti-IgE or IPSE/alpha-1 alone, or anti-IgE or IPSE/alpha-1 in the presence of LPS. Basophils were cultured in the presence of IL-3, a survival factor for these cells. Intact functionality of the basophils was controlled by stimulation with ionomycin that induces maximal cytokine release. LPS differentially affected basophil IL-4 and IL-13 release: The IL-4 release induced by LPS was low compared to that induced by anti-IgE, while the IL-13 release induced by LPS was comparable to that induced by anti-IgE (Figures 8A,B). Simultaneous stimulation of basophils with LPS (via TLR4) and anti-IgE or IPSE/alpha-1 (*via* FcεRI-bound IgE) enhanced both the IL-4 and IL-13 release induced by anti-IgE or IPSE/alpha-1 alone (Figures 9A–D). Boosted IL-4 and IL-13 release from anti-IgE-stimulated basophils—although to a lesser extent—was also observed when basophils were co-stimulated with the TLR2/6 agonist di-acyl lipopeptide FSL-1 (Pam2CGDPKHPKSF) (data not shown). This suggests that basophils stimulated simultaneously by both, FcεRI and TLR, can provide very high local IL-4 and IL-13 concentrations.

### IPSE/alpha-1-induced basophil IL-4 and IL-13 inhibited pro-inflammatory cytokine release from LPS-activated monocytes in the context of whole PBMC

**Figure 10:**
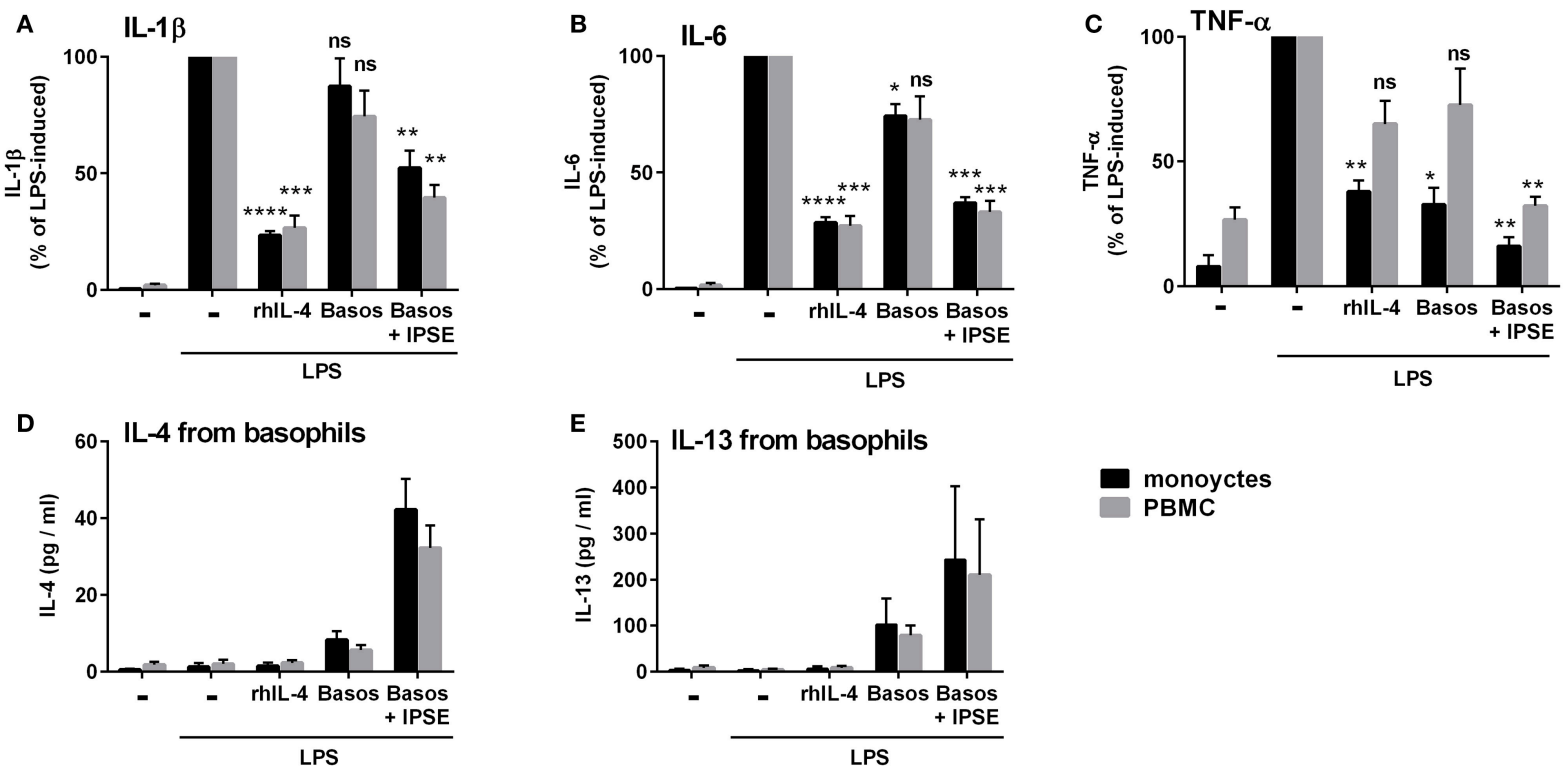
IPSE/alpha-1-triggered basophil IL-4 inhibited the release of IL-1β **(A)**, IL-6 **(B)**, and TNF-α **(C)** from LPS-activated monocytes in the context of whole PBMC. Inhibition correlated with IL-4 **(D)** and IL-13 **(E)** release from basophils. The experiment shown in Figure 1 was repeated with PBMC instead of purified monocytes. Data for PBMC are presented as mean ± SEM, *n* = 4; (TNF-α: *n* = 3). Statistics were performed with the paired *t*-test; significance was shown vs. LPS without additions; ns, not significant, ^*^p < 0.05, ^**^p < 0.01, ^***^p < 0.001, and ^****^p < 0.0001.

*In vivo* schistosome egg granulomas consist of a variety of immune cells that are also found in whole peripheral blood mononuclear cells (PBMC), specifically B cells, T cells, and eosinophils (4). We therefore investigated whether IPSE/alpha-1-triggered basophil IL-4/IL-13 inhibits pro-inflammatory cytokine release (IL-1β, IL-6 and TNF-α) from monocytes in the context of PBMC. We found that, similar to the monocyte-basophil co-culture, pro-inflammatory cytokine production was significantly inhibited in the presence of IPSE/alpha-1 and basophils. The inhibitory capacity correlated with IL-4/IL-13 release from IPSE/alpha-1-triggered basophils (Figure 10). Thus, IPSE/alpha-1 has the potential to control inflammation during *S. mansoni* infection *via* basophil-IL-4 both in the context of isolated monocytes and whole human PBMC.

## Discussion

Here, we demonstrated that IL-4/IL-13 produced by IPSE/alpha-1-stimulated basophils inhibited the release of the pro-inflammatory cytokines IL-1β, IL-6 and TNF-α from LPS-activated monocytes. LPS-activated monocytes cultured in the presence of IPSE/alpha-1-stimulated basophils or their supernatants developed a phenotype similar to AAMs, with elevated expression of CD206 (mannose receptor) and CD209 (DC-SIGN). Using specific antibodies, both IPSE/alpha-1 and basophils were detected in the granuloma around schistosome eggs in sections of liver and gut of infected mice, indicating that basophils come into close contact with IPSE/alpha-1. Importantly, TLR ligation (which occurs when bacterial products enter the host *via* egg-induced gut lesions) boosted the IPSE/alpha-1-induced IL-4/IL-13 release from basophils, thus providing high local IL-4/IL-13 concentrations. Finally, IPSE/alpha-1 also had an anti-inflammatory effect when basophils were co-cultured with whole PBMC instead of purified monocytes.

The eggs of *S. mansoni* are responsible for the clinical impact of schistosomiasis by inducing granulomatous inflammation in liver and intestinal tissues (3). While migrating through the tissue to reach the gut lumen, the eggs exert both pro- and anti-inflammatory effects. Inflammation is necessary to get the eggs out of the host to complete the life cycle of this parasite (5, 26). However, open migration channels left by eggs that penetrate the gut wall are invaded by gut microbiota (and their products), resulting in excessive inflammation if not strictly controlled. The hallmark of the immune response toward schistosomes is the induction of a Th2 response during acute schistosomiasis, which shifts during the chronic infection phase toward a modified or regulated Th2 response characterized by regulatory T and B cells and AAMs (27). Responsible for Th2 induction as well as subsequent immunosuppression are the schistosome eggs, which secrete immunomodulatory factors (4). There are varying informations on the number of excretory/secretory products of schistosome eggs in the literature ranging from 188 to five (28). According to Mathieson et al. (14) and our own unpublished findings, schistosome eggs release only few excretory/secretory proteins with different isoforms at notable amounts—these include the glycoproteins IPSE/alpha-1 and omega-1. IPSE/alpha-1 had been shown to induce IL-4 and IL-13 release from basophils (16), while omega-1 conditions dendritic cells to polarize naïve T helper cells toward Th2 (22). Notably, IPSE/alpha-1 represents more than 80% of the egg secretions (14) underlining its importance for host parasite interaction. Depletion experiments with antibodies to IPSE/alpha-1 have shown, that IPSE/alpha-1 is the only compound in egg secretions and even in whole extract of schistosome eggs (SmEA) able to trigger basophils for IL-4 and IL-13 release (16, 17).

IL-4 and IL-13 are well known as Th2 key cytokines, and naïve T helper cells develop in the presence of IL-4 into Th2 cells (29, 30). Thus, the basophil IL-4-inducing activity of IPSE/alpha-1 makes this molecule a very likely candidate for Th2 induction in schistosome infection (31). Indeed, recently, a number of studies have demonstrated that basophils are able to induce Th2 responses (32–34). However, the Th2-inducing ability of basophils could not be confirmed by others (35), and also the basophil IL-4/IL-13 production triggered by IPSE/alpha-1 failed to induce a Th2 response *in vitro* or *in vivo* (15, 36). In contrast, omega-1, the second well characterized egg-secreted glycoprotein, has been shown to initiate an IL-4-independent Th2 response *via* conditioning of dendritic cells *in vitro* and *in vivo*, while IPSE/alpha-1 did not (36, 37). These data suggest that IPSE/alpha-1-triggered basophil IL-4 production is not involved in Th2 induction.

Thus, we asked, what could be the function of IPSE/alpha-1-induced basophil-derived IL-4 and IL-13? Several studies have demonstrated the protective role of AAMs in schistosomiasis (38, 39), and that they are induced by IL-4 and IL-13 (40). However, the source of these two cytokines is still a matter of debate. A recent study reported that Th2 cells are sufficient, and that basophils are not needed for protection during schistosome infection (41). However, other studies have shown that IL-4 from FcεRI+, non-B, non-T cells are responsible for protection (42) and that basophils are the major source for IL-4 (11, 12). These contradictory outcomes might result from different knock-out mouse models in combination with different parasite infection models (intestinal vs. tissue-dwelling parasites).

The relevance of basophil-derived IL-4 in inflammation control got further support by a recent *in vitro* study showing that basophil IL-4 and IL-13 promote alternative activation of human monocytes (43). Moreover, in a mouse model of chronic IgE-mediated skin allergy, IL-4 from basophils recruited to inflamed skin converted inflammatory into anti-inflammatory M2-macrophages (44, 45), while depletion of basophils exaggerated disease in mouse models for autoimmune colitis (46) as well as for multiple sclerosis [experimental autoimmune encephalomyelitis (EAE)] (47).

The traditional view of basophils as pro-inflammatory effector cells providing mediators (histamine) and cytokines (IL-4/IL-13) to induce and/or amplify a Th2 response in parasite infection and allergy has been further questioned recently by studies showing that after binding to the H2 receptor, histamine enhanced anti-inflammatory IL-10 production by dendritic cells (48). Moreover, depending on the cytokine environment (IL-3 vs. TSLP), basophils have been shown to preferentially release either histamine or IL-4 (49), and that TLR-activated basophils release Th2 cytokines but do not degranulate (50). Together, the data imply a new role for basophils as potential source of IL-4/IL-13 for induction of an anti-inflammatory immune response and, thus, a paradigm shift from basophils as purely inflammatory effector cells to anti-inflammatory, wound-healing, resolution phase cells.

The glycoprotein IPSE/alpha-1, secreted from live schistosome eggs, triggers the release of IL-4/IL-13 from basophils, suggesting that this molecule is the link between schistosome eggs and basophil-released IL-4/IL-13 for the induction of AAMs and inflammation control in schistosome infection. A recent study supported a potential anti-inflammatory role of IPSE/alpha-1. H-IPSE, a homologous molecule in *S. haematobium* (23), was used for treatment of ifosfamide-induced hemorrhagic cystitis (IHC) in mice (24). Administration of H-IPSE reduced IHC-induced bladder hemorrhage in an IL-4-dependent manner. Thus, the basophil IL-4-inducing activity of IPSE/alpha-1 and homologs in other schistosome species might open up new directions for therapy of inflammatory diseases.

The capacity of IL-4 to activate anti-inflammatory macrophages (that possess wound-healing and resolution phase properties) has been used to enhance repair and functional recovery after spinal cord injury by a single intraspinal injection of IL-4 (51, 52) and to improve integration of implants coated with an IL-4-eluting matrix (52). Noteworthy, IL-4 and IL-13 exert a number of anti-inflammatory functions, in addition to alternative activation of macrophages (53, 54), including inhibition of pro-inflammatory cytokine release from LPS-activated monocytes, such as IL-1β, IL-6 and TNF-α (55) and inhibition of Th17 responses (55–57).

In the present study we showed that IPSE/alpha-1-induced basophil IL-4 inhibited pro-inflammatory cytokine release from LPS-activated monocytes, and moreover, that monocytes co-cultured with IPSE/alpha-stimulated basophils developed an AAM-like phenotype. For human AAMs, especially in the context of schistosome granulomas, the mannose receptor CD206 has been proposed as a characteristic marker (58, 59). This was supported by our data: culturing human LPS-activated monocytes in the presence of IL-4-containing basophil supernatant increased the expression of CD206-positive monocytes from two to 11%. Similarly, the percentage of CD209 (DC-SIGN)-positive monocytes increased from 0.8 up to 22%, suggesting CD209 is also a suitable marker for human AAMs. We did not detect other proposed surface markers for human AAMs on *in vitro*-generated human AAMs, such as CD163 and CD200R (although CD200R was clearly detectable on the surface of basophils) (60–62).

Immunohistology was performed on schistosome-infected tissue to address the question whether IPSE/alpha-1 meets basophils *in vivo*, i.e., in the granuloma. Since samples from infected humans were not available, immunohistology with specific antibodies to IPSE/alpha-1 and basophils, respectively, was performed on liver and gut from infected mice. Although there are differences in the pathology of schistosomiasis between humans and mice (a. o. due to the difference in host/parasite size ratio and, thus, the relative worm load), the mouse model is widely used and accepted as a model for investigation of immunology of schistosomiasis (63).

Expression of IPSE/alpha-1 correlated with the maturation stage of the eggs: immature, freshly embolized eggs surrounded by only few recruited inflammatory cells and intact liver parenchyma (early granuloma formation) did usually not yet stain for IPSE/alpha-1, while IPSE/alpha-1 was detected in larger further developed eggs/granulomas in the typical subshell location. Secreted IPSE/alpha-1 was detected mainly in full-blown granulomas in the immediate vicinity of the eggs. The subshell area of the eggs consists of the von Lichtenberg's envelope, which surrounds the miracidium and contains large amounts of rough endoplasmic reticulum, and the so-called Reynolds' Layer, where excretory/secretory proteins are produced and stored, respectively (23). In the subshell area also omega-1, another egg-secreted glycoprotein, which interacts with dendritic cells, has been located earlier (64).

While the basophil to monocyte ratio of 1:1 in our culture does not reflect the situation in peripheral blood, where basophils represent 0.5–1% and monocytes 2–8% of the leukocytes, we expected that basophils are attracted to and, thus, concentrated in the egg granulomas. Indeed, immunohistology revealed a striking enrichment of basophils in the granulomatous lesions, whereas only few basophils were observed in the residual liver or gut tissue. Of note: control tissue from non-infected mice was nearly void of basophils. In small granulomas basophils were detected close to the eggs, but in large granulomas mainly in the outer rim of the inflammatory lesions and less frequently near the eggs. Noteworthy, the antibody used for basophil staining is directed to mMCP-8, a granule compound of murine basophils that is released during degranulation. Therefore, we think that basophils in close vicinity to mature IPSE/alpha-1-secreting eggs are degranulated upon contact with IPSE/alpha-1 and are, thus, no longer identified as basophils (ghost cells) with the released material being eliminated by diffusion and/or degradation.

Nevertheless the question remained, whether basophils produce enough IL-4 and IL-13 to induce alternative activation of macrophages. Although Th2 cells are present in much higher numbers in blood and tissue, basophils release more IL-4 than Th2 cells on a cellular level (65). Recently, it was reported that basophils express TLRs, and co-stimulation of TLR and FcεRI resulted in higher histamine release compared to FcεRI stimulation alone (66, 67). By RT-PCR and flow cytometry, we confirmed TLR expression on basophils and found that the expression patterns varied between individuals. We, therefore, asked whether TLR activation might likewise enhance IgE/FcεRI-mediated IL-4 and IL-13 release from basophils. Indeed simultaneous stimulation by LPS (or the TLR2/6 agonist FSL-1) boosted the release of IL-4 and IL-13 two- and five-fold, respectively. This suggests that the high number of basophils within schistosome egg granulomas together with the boosted IL-4 and IL-13 release in the context of TLR activation will result in a local IL-4 and IL-13 concentration high enough to induce AAMs.

In conclusion, the results of our study led us to the following hypothetical model for inflammation control in schistosome infection: Basophils attracted to schistosome eggs in the gut are stimulated by both IPSE/alpha-1 *via* IgE/FcεRI and by bacterial products *via* TLR. Combined stimulation boosts basophil cytokine release to provide local IL-4/IL-13 concentrations high enough to turn pro-inflammatory monocytes/macrophages into anti-inflammatory AAMs, thereby ensuring survival of the hosts.

## Author contributions

KK, HH, and GS designed the experiments. KK, KL, SN, KV, AK, and MD performed the experiments. KK, KL, and GS analyzed the data. GS, MD, and HH wrote the manuscript.

### Conflict of interest statement

The authors declare that the research was conducted in the absence of any commercial or financial relationships that could be construed as a potential conflict of interest.

## References

[1] Wilson MS Maizels RM. Regulation of allergy and autoimmunity in helminth infection. Clin Rev Allergy Immunol. (2004) 26:35–50. 10.1385/CRIAI:26:1:35 14755074

[2] Wammes LJ Mpairwe H Elliott AM Yazdanbakhsh M. Helminth therapy or elimination: epidemiological, immunological, and clinical considerations. Lancet Infect Dis. (2014) 14:1150–62. 10.1016/S1473-3099(14)70771-6 24981042

[3] Gryseels B Polman K Clerinx J Kestens L. Human schistosomiasis. Lancet Lond Engl. (2006) 368:1106–18. 10.1016/S0140-6736(06)69440-3 16997665

[4] Pearce EJ MacDonald AS. The immunobiology of schistosomiasis. Nat Rev Immunol. (2002) 2:499–511. 10.1038/nri843 12094224

[5] Doenhoff MJ. A role for granulomatous inflammation in the transmission of infectious disease: schistosomiasis and tuberculosis. Parasitology (1997) 115(Suppl.):S113–25. 9571697 10.1017/s0031182097001972

[6] Doenhoff MJ Pearson S Dunne DW Bickle Q Lucas S Bain J. Immunological control of hepatotoxicity and parasite egg excretion in Schistosoma mansoni infections: stage specificity of the reactivity of immune serum in T-cell deprived mice. Trans R Soc Trop Med Hyg. (1981) 75:41–53. 10.1016/0035-9203(81)90012-2 6973848

[7] Brunet LR Finkelman FD Cheever AW Kopf MA Pearce EJ. IL-4 protects against TNF-alpha-mediated cachexia and death during acute schistosomiasis. J Immunol. (1997) 159:777–85. 9218595

[8] Herbert DR Hölscher C Mohrs M Arendse B Schwegmann A Radwanska M. Alternative macrophage activation is essential for survival during schistosomiasis and downmodulates T helper 1 responses and immunopathology. Immunity (2004) 20:623–35. 10.1016/S1074-7613(04)00107-4 15142530

[9] Herbert DR Orekov T Perkins C Rothenberg ME Finkelman FD. IL-4R alpha expression by bone marrow-derived cells is necessary and sufficient for host protection against acute schistosomiasis. J Immunol. (2008) 180:4948–55. 10.4049/jimmunol.180.7.4948 18354220 PMC2921971

[10] Anyan W Kumagai T Shimugawara RF Seki T Akao N Obata K Schistosome eggs have a direct role in the induction of baosphils capable of a high level of IL-4 production: comparative study of single- and bisexual infection of Schistosoma mansoni *in vivo*. Trop Med Health (2010) 28:13–22. 10.2149/tmh.2009-24

[11] Mitre E Taylor RT Kubofcik J Nutman TB. Parasite antigen-driven basophils are a major source of IL-4 in human filarial infections. J Immunol. (2004) 172:2439–45. 10.4049/jimmunol.172.4.2439 14764715

[12] van Panhuys N Prout M Forbes E Min B Paul WE Le Gros G. Basophils are the major producers of IL-4 during primary helminth infection. J Immunol. (2011) 186:2719–28. 10.4049/jimmunol.1000940 21270410 PMC3488853

[13] Schramm G Haas H. Th2 immune response against Schistosoma mansoni infection. Microbes Infect. (2010) 12:881–8. 10.1016/j.micinf.2010.06.001 20542135

[14] Mathieson W Wilson RA. A comparative proteomic study of the undeveloped and developed Schistosoma mansoni egg and its contents: the miracidium, hatch fluid and secretions. Int J Parasitol. (2010) 40:617–28. 10.1016/j.ijpara.2009.10.014 19917288

[15] Schramm G Mohrs K Wodrich M Doenhoff MJ Pearce EJ Haas H. Cutting edge: IPSE/alpha-1, a glycoprotein from Schistosoma mansoni eggs, induces IgE-dependent, antigen-independent IL-4 production by murine basophils *in vivo*. J Immunol. (2007) 178:6023–27. 10.4049/jimmunol.178.10.6023 17475824

[16] Schramm G Falcone FH Gronow A Haisch K Mamat U Doenhoff MJ. Molecular characterization of an interleukin-4-inducing factor from Schistosoma mansoni eggs. J Biol Chem. (2003) 278:18384–92. 10.1074/jbc.M300497200 12624091

[17] Meyer NH Mayerhofer H Tripsianes K Blindow S Barths D Mewes A. A crystallin fold in the interleukin-4-inducing principle of schistosoma mansoni eggs (IPSE/α-1) mediates IgE binding for antigen-independent basophil activation. J Biol Chem. (2015) 290:22111–26. 10.1074/jbc.M115.675066. 26163514 PMC4571962

[18] Falcone FH Dahinden CA Gibbs BF Noll T Amon U Hebestreit H. Human basophils release interleukin-4 after stimulation with Schistosoma mansoni egg antigen. Eur J Immunol. (1996) 26:1147–55. 8647180 10.1002/eji.1830260528

[19] Kaur I Schramm G Everts B Scholzen T Kindle KB Beetz C Interleukin-4-inducing principle from Schistosoma mansoni eggs contains a functional C-terminal nuclear localization signal necessary for nuclear translocation in mammalian cells but not for its uptake. Infect Immun. (2011) 79:1779–88. 10.1128/IAI.01048-10 21220486 PMC3067533

[20] Wuhrer M Balog CI Catalina MI Jones FM Schramm G Haas H. IPSE/alpha-1, a major secretory glycoprotein antigen from schistosome eggs, expresses the Lewis X motif on core-difucosylated N-glycans. FEBS J. (2006) 273:2276–92. 10.1111/j.1742-4658.2006.05242.x 16650003

[21] Haisch K Gibbs BF Koerber H Ernst M Grage-Griebenow E Schlaak M. Purification of morphologically and functionally intact human basophils to near homogeneity. J Immunol Methods (1999) 226:129–37. 10410978 10.1016/s0022-1759(99)00059-9

[22] Smith P Fallon RE Mangan NE Walsh CM Saraiva M Sayers JR McKenzie ANJ. Schistosoma mansoni secretes a chemokine binding protein with antiinflammatory activity. J Exp Med. (2005) 202:1319–25. 10.1084/jem.20050955 16301741 PMC2212990

[23] Pennington LF Alouffi A Mbanefo EC Ray D Heery DM Jardetzky TS H-IPSE is a pathogen-secreted host nucleus infiltrating protein (infiltrin) expressed exclusively by the Schistosoma haematobium egg stage. Infect Immun. (2017) 85:1–14. 10.1128/IAI.00301-17 PMC5695104 28923894

[24] Mbanefo EC Le L Pennington LF Odegaard JI Jardetzky TS Alouffi A. Therapeutic exploitation of IPSE, a urogenital parasite-derived host modulatory protein, for chemotherapy-induced hemorrhagic cystitis. FASEB J. (2018) 32:4408–19. 10.1096/fj.201701415R 29613835 PMC6044057

[25] Brunner T Heusser CH Dahinden CA. Human peripheral blood basophils primed by interleukin 3 (IL-3) produce IL-4 in response to immunoglobulin E receptor stimulation. J Exp Med. (1993) 177:605–11. 10.1084/jem.177.3.605 8436904 PMC2190932

[26] Hams E Aviello G Fallon PG The schistosoma granuloma: friend or foe? Front Immunol. (2013) 4:89 10.3389/fimmu.2013.00089 23596444 PMC3625856

[27] Díaz A Allen JE. Mapping immune response profiles: the emerging scenario from helminth immunology. Eur J Immunol. (2007) 37:3319–26. 10.1002/eji.200737765 18000958

[28] Cass CL Johnson JR Califf LL Xu T Hernandez HJ Stadecker MJ. Proteomic analysis of Schistosoma mansoni egg secretions. Mol Biochem Parasitol. (2007) 155:84–93. 10.1016/j.molbiopara.2007.06.002 17644200 PMC2077830

[29] Mosmann TR Coffman RL. TH1 and TH2 cells: different patterns of lymphokine secretion lead to different functional properties. Annu Rev Immunol. (1989) 7:145–73. 10.1146/annurev.iy.07.040189.001045 2523712

[30] Hsieh CS Heimberger AB Gold JS O'Garra A Murphy KM. Differential regulation of T helper phenotype development by interleukins 4 and 10 in an alpha beta T-cell-receptor transgenic system. Proc Natl Acad Sci USA. (1992) 89:6065–9. 1385868 10.1073/pnas.89.13.6065 PMC49438

[31] Haas H Falcone FH Holland MJ Schramm G Haisch K Gibbs BF. Early interleukin-4: its role in the switch towards a Th2 response and IgE-mediated allergy. Int Arch Allergy Immunol. (1999) 119:86–94. 10.1159/000024182 10394099

[32] Perrigoue JG Saenz SA Siracusa MC Allenspach EJ Taylor BC Giacomin PR. MHC class II-dependent basophil-CD4+ T cell interactions promote T(H)2 cytokine-dependent immunity. Nat Immunol. (2009) 10:697–705. 10.1038/ni.1740 19465906 PMC2711559

[33] Sokol CL Chu N-Q Yu S Nish SA Laufer TM Medzhitov R. Basophils function as antigen-presenting cells for an allergen-induced T helper type 2 response. Nat Immunol. (2009) 10:713–20. 10.1038/ni.1738 19465907 PMC3252751

[34] Yoshimoto T Yasuda K Tanaka H Nakahira M Imai Y Fujimori Y. Basophils contribute to T(H)2-IgE responses *in vivo* via IL-4 production and presentation of peptide-MHC class II complexes to CD4+ T cells. Nat Immunol. (2009) 10:706–12. 10.1038/ni.1737 19465908

[35] Hammad H Plantinga M Deswarte K Pouliot P Willart MAM Kool M. Inflammatory dendritic cells–not basophils–are necessary and sufficient for induction of Th2 immunity to inhaled house dust mite allergen. J Exp Med. (2010) 207:2097–111. 10.1084/jem.20101563 20819925 PMC2947072

[36] Everts B Perona-Wright G Smits HH Hokke CH van der Ham AJ Fitzsimmons CM. Omega-1, a glycoprotein secreted by Schistosoma mansoni eggs, drives Th2 responses. J Exp Med. (2009) 206:1673–80. 10.1084/jem.20082460 19635864 PMC2722183

[37] Everts B Hussaarts L Driessen NN Meevissen MHJ Schramm G van der Ham AJ. Schistosome-derived omega-1 drives Th2 polarization by suppressing protein synthesis following internalization by the mannose receptor. J Exp Med. (2012) 209:1753–67, S1. 10.1084/jem.20111381 22966004 PMC3457738

[38] Chen F Liu Z Wu W Rozo C Bowdridge S Millman A. An essential role for TH2-type responses in limiting acute tissue damage during experimental helminth infection. Nat Med. (2012) 18:260–6. 10.1038/nm.2628 22245779 PMC3274634

[39] Gause WC Wynn TA Allen JE. Type 2 immunity and wound healing: evolutionary refinement of adaptive immunity by helminths. Nat Rev Immunol. (2013) 13:607–14. 10.1038/nri3476 23827958 PMC3789590

[40] Gordon S Martinez FO. Alternative activation of macrophages: mechanism and functions. Immunity (2010) 32:593–604. 10.1016/j.immuni.2010.05.007 20510870

[41] Schwartz C Turqueti-Neves A Hartmann S Yu P Nimmerjahn F Voehringer D. Basophil-mediated protection against gastrointestinal helminths requires IgE-induced cytokine secretion. Proc Natl Acad Sci USA. (2014) 111:E5169–77. 10.1073/pnas.1412663111 25404305 PMC4260590

[42] Jankovic D Kullberg MC Dombrowicz D Barbieri S Caspar P Wynn TA. Fc epsilonRI-deficient mice infected with Schistosoma mansoni mount normal Th2-type responses while displaying enhanced liver pathology. J Immunol. (1997) 159:1868–75. 9257851

[43] Borriello F Longo M Spinelli R Pecoraro A Granata F Staiano RI. IL-3 synergises with basophil-derived IL-4 and IL-13 to promote the alternative activation of human monocytes. Eur J Immunol. (2015) 45:2042–51. 10.1002/eji.201445303 25824485 PMC4496336

[44] Egawa M Mukai K Yoshikawa S Iki M Mukaida N Kawano Y. Inflammatory monocytes recruited to allergic skin acquire an anti-inflammatory M2 phenotype via basophil-derived interleukin-4. Immunity (2013) 38:1–11. 10.1016/j.immuni.2012.11.014 23434060

[45] Obata-Ninomiya K Ishiwata K Tsutsui H Nei Y Yoshikawa S Kawano Y. The skin is an important bulwark of acquired immunity against intestinal helminths. J Exp Med. (2013) 210:2583–95. 10.1084/jem.20130761 24166714 PMC3832932

[46] Gomez MR Talke Y Hofmann C Ketelsen I Hermann F Reich B. Basophils control T-cell responses and limit disease activity in experimental murine colitis. Mucosal Immunol. (2014) 7:188–99. 10.1038/mi.2013.38 23757302

[47] Musio S Costanza M Poliani PL Fontana E Cominelli M Abolafio G. Treatment with anti-FcεRIα antibody exacerbates EAE and T-cell immunity against myelin. Neurol Neuroimmunol Neuroinflamm. (2017) 4:e342. 10.1212/NXI.0000000000000342 28616446 PMC5462602

[48] Frei R Ferstl R Konieczna P Ziegler M Simon T Rugeles TM. Histamine receptor 2 modifies dendritic cell responses to microbial ligands. J Allergy Clin Immunol. (2013) 132:194–204. 10.1016/j.jaci.2013.01.013 23465664

[49] Siracusa MC Saenz SA Hill DA Kim BS Headley MB Doering TA. TSLP promotes interleukin-3-independent basophil haematopoiesis and type 2 inflammation. Nature (2011) 477:229–33. 10.1038/nature10329 21841801 PMC3263308

[50] Suurmond J Stoop JN Rivellese F Bakker AM Huizinga TWJ Toes REM. Activation of human basophils by combined toll-like receptor- and FcepsilonRI-triggering can promote Th2 skewing of naive T helper cells. Eur J Immunol. (2014) 44:386–96. 10.1002/eji.201343617 24122358

[51] Francos-Quijorna I Amo-Aparicio J Martinez-Muriana A López-Vales R. IL-4 drives microglia and macrophages toward a phenotype conducive for tissue repair and functional recovery after spinal cord injury. Glia (2016) 64:2079–92. 10.1002/glia.23041 27470986

[52] Hachim D LoPresti ST Yates CC Brown BN. Shifts in macrophage phenotype at the biomaterial interface via IL-4 eluting coatings are associated with improved implant integration. Biomaterials (2017) 112:95–107. 10.1016/j.biomaterials.2016.10.019 27760399 PMC5121003

[53] Luzina IG Keegan AD Heller NM Rook GAW Shea-Donohue T Atamas SP. Regulation of inflammation by interleukin-4: a review of “alternatives”. J Leukoc Biol. (2012) 92:753–64. 10.1189/jlb.0412214 22782966 PMC3441310

[54] Seki T Kumagai T Kwansa-Bentum B Furushima-Shimogawara R Anyan WK Miyazawa Y. Interleukin-4 (IL-4) and IL-13 suppress excessive neutrophil infiltration and hepatocyte damage during acute murine schistosomiasis japonica. Infect Immun. (2012) 80:159–68. 10.1128/IAI.05581-11 22038918 PMC3255664

[55] te Velde AA Huijbens RJ Heije K de Vries JE Figdor CG. Interleukin-4 (IL-4) inhibits secretion of IL-1 beta, tumor necrosis factor alpha, and IL-6 by human monocytes. Blood (1990) 76:1392–7. 2119829

[56] Guenova E Skabytska Y Hoetzenecker W Weindl G Sauer K Tham M. IL-4 abrogates T(H)17 cell-mediated inflammation by selective silencing of IL-23 in antigen-presenting cells. Proc Natl Acad Sci USA. (2015) 112:2163–8. 10.1073/pnas.1416922112 25646481 PMC4343151

[57] He R Kim HY Yoon J Oyoshi MK MacGinnitie A Goya S. Exaggerated IL-17 response to epicutaneous sensitization mediates airway inflammation in the absence of IL-4 and IL-13. J Allergy Clin Immunol. (2009) 124:761–770.e1. 10.1016/j.jaci.2009.07.040 19815118 PMC2895457

[58] Linehan SA Coulson PS Wilson RA Mountford AP Brombacher F Martinez-Pomares L IL-4 receptor signaling is required for mannose receptor expression by macrophages recruited to granulomata but not resident cells in mice infected with Schistosoma mansoni. Lab Investig J Tech Methods Pathol. (2003) 83:1223–31. 10.1097/01.LAB.0000081392.93701.6F 12920251

[59] Paveley RA Aynsley SA Turner JD Bourke CD Jenkins SJ Cook PC. The Mannose Receptor (CD206) is an important pattern recognition receptor (PRR) in the detection of the infective stage of the helminth Schistosoma mansoni and modulates IFNgamma production. Int J Parasitol. (2011) 41:1335–45. 10.1016/j.ijpara.2011.08.005 22036898

[60] Ambarus CA Krausz S van Eijk M Hamann J Radstake TRDJ Reedquist KA. Systematic validation of specific phenotypic markers for *in vitro* polarized human macrophages. J Immunol Methods (2012) 375:196–206. 10.1016/j.jim.2011.10.013 22075274

[61] Barros MHM Hauck F Dreyer JH Kempkes B Niedobitek G. Macrophage polarisation: an immunohistochemical approach for identifying M1 and M2 macrophages. PloS One (2013) 8:e80908. 10.1371/journal.pone.0080908 24260507 PMC3829941

[62] Koning N van Eijk M Pouwels W Brouwer MSM Voehringer D Huitinga I. Expression of the inhibitory CD200 receptor is associated with alternative macrophage activation. J Innate Immun. (2010) 2:195–200. 10.1159/000252803 20375636

[63] Fallon PG. Immunopathology of schistosomiasis: a cautionary tale of mice and men. Immunol Today (2000) 21:29–35. 10.1016/S0167-5699(99)01551-0 10637556

[64] Fitzsimmons CM Schramm G Jones FM Chalmers IW Hoffmann KF Grevelding CG. Molecular characterization of omega-1: a hepatotoxic ribonuclease from Schistosoma mansoni eggs. Mol Biochem Parasitol. (2005) 144:123–7. 10.1016/j.molbiopara.2005.08.003 16143411

[65] Kasaian MT Clay MJ Happ MP Garman RD Hirani S Luqman M. IL-4 production by allergen-stimulated primary cultures: identification of basophils as the major IL-4-producing cell type. Int Immunol. (1996) 8:1287–97. 10.1093/intimm/8.8.1287 8918698

[66] Bieneman AP Chichester KL Chen Y-H Schroeder JT. Toll-like receptor 2 ligands activate human basophils for both IgE-dependent and IgE-independent secretion. J Allergy Clin Immunol. (2005) 115:295–301. 10.1016/j.jaci.2004.10.018 15696084

[67] Komiya A Nagase H Okugawa S Ota Y Suzukawa M Kawakami A. Expression and function of toll-like receptors in human basophils. Int Arch Allergy Immunol. (2006) 140(Suppl. 1):23–7. 10.1159/000092707 16772723

